# Comparison of Very Low Energy Diet Products Available in Australia and How to Tailor Them to Optimise Protein Content for Younger and Older Adult Men and Women

**DOI:** 10.3390/healthcare4030071

**Published:** 2016-09-21

**Authors:** Alice A. Gibson, Janet Franklin, Andrea L. Pattinson, Zilvia G. Y. Cheng, Samir Samman, Tania P. Markovic, Amanda Sainsbury

**Affiliations:** 1 The Boden Institute of Obesity, Nutrition, Exercise & Eating Disorders, Sydney Medical School, The University of Sydney, Sydney 2006, Australia; andrea.pattinson@sydney.edu.au (A.L.P.); zche8565@uni.sydney.edu.au (Z.G.Y.C.); tania.markovic@sydney.edu.au (T.P.M.); amanda.salis@sydney.edu.au (A.S.); 2 Charles Perkins Centre, The University of Sydney, Sydney 2006, Australia; 3 Metabolism & Obesity Services, Royal Prince Alfred Hospital, Camperdown 2050, Australia; janet.franklin@sswahs.nsw.gov.au; 4 Department of Human Nutrition, University of Otago, Dunedin 9054, New Zealand; samir.samman@otago.ac.nz; 5 School of Life and Environmental Sciences, The University of Sydney, Sydney 2006, Australia

**Keywords:** obesity, weight loss, diet reducing, nutritional requirements, dietary protein

## Abstract

Very low energy diets (VLED) are efficacious in inducing rapid weight loss but may not contain adequate macronutrients or micronutrients for individuals with varying nutritional requirements. Adequate protein intake during weight loss appears particularly important to help preserve fat free mass and control appetite, and low energy and carbohydrate content also contributes to appetite control. Therefore, the purpose of this study was to compare the nutritional content (with a focus on protein), nutritional adequacy and cost of all commercially-available VLED brands in Australia. Nutritional content and cost were extracted and compared between brands and to the Recommended Dietary Intake (RDI) or adequate intake (AI) of macronutrients and micronutrients for men and women aged 19–70 years or >70 years. There was wide variability in the nutritional content, nutritional adequacy and cost of VLED brands. Most notably, even brands with the highest daily protein content, based on consuming three products/day (KicStart™ and Optislim^®^, ~60 g/day), only met estimated protein requirements of the smallest and youngest women for whom a VLED would be indicated. Considering multiple options to optimise protein content, we propose that adding pure powdered protein is the most suitable option because it minimizes additional energy, carbohydrate and cost of VLEDs.

## 1. Introduction

While lifestyle modifications remain the cornerstone of obesity treatment, adjunct therapies such as very low energy diets (VLEDs, also called very low calorie diets or VLCDs), pharmacotherapy and bariatric surgery are indicated for certain individuals [1,2,3]. VLEDs are the most intensive form of dietary intervention for obesity. They involve severely restricting energy intake to less than 3350 kJ per day with the use of three specially formulated meal replacement products that replace all usual food intake [4]. This severe energy restriction results in substantial and rapid weight losses of between 1.5 and 2.5 kg per week, typically over periods of 8–16 weeks [5]. As such, they are the single most effective dietary intervention for obesity over the short term. A recent study showed that a person commencing a VLED has an ~80% chance of losing ≥12.5% of their initial body weight, compared to only ~50% of people commencing a diet involving moderate energy restriction (that is, diet in which energy intake is restricted by ~2000 kJ per day) [6]. Furthermore, a recent meta-analysis showed that a VLED with behavioural therapy, compared with a behavioural intervention alone, resulted in an additional weight loss of 3.9 kg at 12 months [7]. In contrast to many of the weight loss products available on the market today, VLEDs are backed by decades of medical research and have been in clinical use for almost 40 years.

Despite the efficacy and medical research behind VLEDs, they may be underutilised by clinicians. A 2008 survey of Australian dietitians revealed that only 1.5% of respondents reported prescribing a rapid weight loss program to their clients [8]. This lack of clinical use of VLEDs may be related to the fact that in the late 1970s, one of the first commercially-available VLED brands caused 17 deaths after 8–26 weeks of use [9,10]. These deaths were attributed to the use in that VLED brand of a low quality protein deficient in essential amino acids, as well as the absence of any fortification with vitamins or minerals [9,10]. Therefore, while modern formulations of VLEDs are based on high quality complete protein and are considered safe [4], they may be underutilised.

In addition to concerns about safety, possible underutilisation of VLEDs could be related to fears about rapid weight regain and large losses of fat free mass, with fat free mass being an important determinant of energy expenditure [11]. Among clinicians and the lay public alike, it is commonly believed that it is better to lose weight gradually than rapidly, perhaps in part because rapid weight loss supposedly does not allow time for new healthy eating and physical activity habits to be formed [12,13,14]. However, this assertion of rapid regain following rapid weight loss is not support by scientific evidence [13,14]. Not only has greater initial weight loss been shown to be predictive of long term success in maintaining a lower body weight [12,15,16,17], but studies comparing different rates of weight loss (with equal total amounts of weight loss) have demonstrated no difference in weight regain, regardless of whether dietary advice is provided post-intervention [6,18] or not [19]. This is not to say that an individual will not regain weight after rapid weight loss, but more so that concerns about rapid weight regain solely due to the rate of weight loss are unfounded. On the other hand, concerns about losses of fat free mass with rapid weight loss have some scientific support. A meta-analysis showed that loss of fat free mass during weight loss was positively correlated with the degree of energy restriction used to achieve weight loss (*r*^2^ = 0.31, *p =* 0.006) [20], supporting the hypothesis that the rate of weight loss is associated with a greater loss of fat free mass. Further, a recent study [19] compared an eight-week VLED (2100 kJ per day) with a 17-week low energy diet (LED; 5200 kJ per day), and showed that the VLED group lost a greater proportion of weight as fat free mass compared with the LED. Moreover, the proportion of weight lost as fat free mass was associated with weight regain in both groups at nine months follow up, despite no difference in weight regain between the groups. In summary, despite lack of evidence that VLEDs lead to greater weight regain than moderately energy-restricted diets, they may result in greater loss of fat free mass.

There is strong and consistent evidence that loss of fat free mass during weight loss can be attenuated by diets providing protein intakes of ≥0.8 g/kg body weight/day (approximately equivalent to the recommend dietary intake [RDI] in Australia for adults aged < 70 years), compared with lower protein diets (<0.8 g/kg/day) [11,21,22,23,24,25,26,27,28,29,30,31,32,33,34,35]. This level of protein intake has also been shown to result in greater energy expenditure and satiety during weight loss, relative to lower protein diets [11,21,22,23,24,25,26,27,28,29,30,31,32,33,34,35], albeit research to date has predominantly focused on moderately energy-restricted diets (that is, diets in which energy intake is restricted by ~30%–40% relative to estimated weight maintenance requirements), and two key meta-analyses examining the effect of diet composition on weight loss have specifically excluded VLEDs [34,35]. Given that the rate of weight loss may be associated with the proportion of weight lost as fat free mass, as highlighted above, and that a higher protein intake is associated not only with attenuated loss of fat free mass but also greater energy expenditure and satiety, the protein content of severely energy-restricted diets (e.g., VLEDs) would thus appear to be of even greater importance than that of moderately energy-restricted diets.

Preventing loss of fat free mass during weight loss is of particular importance in older people, in order to avoid exacerbating age-related losses of fat free mass (sarcopenia), which can lead to profound debility and impaired quality of life [36]. This is particularly important in the case of sarcopenic obesity (low fat free mass despite body mass index [BMI] being in the obese range), which is difficult to diagnose in routine clinical practice [37] but may be even more debilitating than either sarcopenia or obesity alone [38]. Older people (aged > 70 years) have increased protein requirements due to an elevated anabolic resistance to ingested protein [39,40], making adequate protein intake during a VLED of even greater significance in older populations. The safety of intentional weight loss in older people is controversial [41,42], and VLEDs are specifically contraindicated for people of ‘advanced age’ [3]. However, the prevalence of obesity is increasing in parallel with an ageing population [43], and obesity is a major cause of disability associated with reduced quality of life and increased frailty [44,45]. As such, clinicians will increasingly be faced with decisions about appropriate weight management for older adults, considering potential benefits of weight loss treatments against potential harms such as exacerbating age-related loss of fat free mass. However, in light of the effectiveness of VLEDs for the treatment of obesity, and with an increasing number of older adults requiring effective obesity treatments, VLEDs could be a suitable option for this population, with due consideration to their increased protein requirements.

In addition to the protein content of VLEDs, their carbohydrate and energy content are also important considerations. Low carbohydrate intake during a VLED is thought to be important for ketosis and appetite suppression, and low energy intake is important for rapid weight loss. Ketosis results when carbohydrate (glucose) is in short supply in the body, leading to increased circulating concentrations of ketones (β-hydroxybutyrate, acetoacetate and acetone), which are produced in the liver by ß-oxidation of free fatty acids. Ketosis is thought to be a key factor in helping to prevent a compensatory increase in the drive to eat despite the severely restricted energy intake of a VLED [46], allowing individuals to lose weight rapidly without feeling excessively hungry. Whilst the exact level of carbohydrate intake at which ketosis occurs is not known, and is likely to vary between individuals due to a variety of factors, ketosis is thought to be unlikely when carbohydrate intake is above 100 g per day [47,48]. Our recent meta-analysis showed that a compensatory increase in appetite was not seen in VLEDs that contained approximately 50 g of carbohydrate (with fasting β-hydroxybutyrate levels of 0.5 mM) [46]. Therefore, a VLED that provides a higher protein content, in addition to a lower carbohydrate and energy content, may aid adherence via reduced appetite, without compromising the rate of weight loss.

VLEDs are generally prescribed in a “one size fits all” approach, whereby individuals replace three meals per day and all other dietary energy sources with three VLED products. However, there is considerable variability in the age and body mass index (BMI) of clinical populations for which VLEDs are indicated. Therefore, a universal approach to VLED prescription is not ideal, as it results in individuals with widely varying dietary protein requirements receiving the same quantity of protein. In addition to potential mismatches between dietary protein requirements and protein content in VLEDs that are prescribed uniformly, there may be considerable variability in the nutritional content of VLED products available, as the nutritional content of VLEDs is currently unregulated. VLEDs are explicitly excluded from two of the most relevant standards of the Food Standards Australia and New Zealand (FSANZ) code (Standard 2.9.3: Formulated meal replacement and formulated supplementary foods, and Standard 2.9.5: Food for special medical purposes) [49]. We propose that the aim of VLED clinical treatment protocols should be to meet protein (and micronutrient) requirements at the lowest carbohydrate and energy level, in order to help preserve fat free mass and control appetite, while still allowing for rapid weight loss. As obesity disproportionately affects those of lower socioeconomic status [50], cost may also be a barrier, so achieving the above within minimal cost is also important. Therefore, the aim of this study is to compare the nutritional content, nutritional adequacy (with a focus on protein) and cost of all commercially-available VLED brands in Australia, and to demonstrate ways in which these factors could be optimised, in order to guide clinicians on how to use and tailor VLEDs effectively for individual clients with varying nutritional requirements.

## 2. Materials and Methods

### 2.1. Identification of VLED Brands in Australia

Brands of VLEDs available in Australia were identified from several sources. Firstly, we identified all pharmacies within a 15 km radius of the Sydney Central Business District using www.findapharmacy.com.au. Names of the pharmacies and phone numbers were exported, listed alphabetically, and two authors (Alice A. Gibson and Zilvia G. Y. Cheng) telephoned every second pharmacy on the list (*n =* 300). Persons answering the phone were then greeted and asked, “which brands of weight loss shakes does your pharmacy stock?” The term ‘weight loss shakes’ was used as it was thought that not all pharmacy staff would be familiar with the terms VLED or meal replacement, and that this term would thus allow us to determine the full range of available products, from which we would later identify those that were eligible for inclusion in this study. In addition, we searched online pharmacy websites, and Google searches were also performed, using the following keywords ‘very low energy diet’, ‘very low calorie diet’, ‘VLED’, ‘VLCD’, ‘weight loss shake’ and ‘meal replacement’. Online searches were complemented with approximately 24 “in person” visits by Alice A. Gibson and Zilvia G. Y. Cheng to major pharmacy retailers, as well as personal communication [51] by Janet Franklin with specialist obesity clinicians via the Dietitians Association of Australia Obesity interest group email forum.

Once all potentially eligible brands were identified, we excluded products from brands that did not meet our definition of a VLED. To be defined as a VLED, the manufacturer’s instructions had to result in a diet that provided less than 3350 kJ per day and replaced all main meals (i.e., total diet replacement) [4]. Therefore, products that could only be used as partial meal replacements were excluded. We considered excluding one brand, Vita Diet, as the instructions on their website for the “Medical Vita Diet Weight Loss Program” were to consume two meal replacement products per day, with a third meal of protein and vegetables (i.e., partial meal replacement). However, in the information listed for some products (shakes and soups) in the Vita Diet range, the website contained the statement “Can be used a VLCD (Very Low Calorie Diet) shake”. Therefore we decided to include this brand as this statement suggests to a clinician or client that the products are suitable as a total diet replacement.

### 2.2. Data Extraction and Analysis

The following information was collected from the websites and/or product packages of eligible brands: availability and cost in Australian dollars (Recommended Retail Price, RRP, of largest quantity available) of all product types and flavours, preparation instructions, suitability with special dietary requirements (i.e., gluten free, lactose free, artificial sweeteners), and macronutrient and micronutrient content per serving. Where information about a particular nutrient was missing, it was assumed that the product did not contain that nutrient. For products that specified that they needed to be mixed with skim milk (versus water), we used the nutrient content values that included skim milk. For products that could be mixed with either skim milk or water, we used the nutrient content of the product as mixed with water.

### 2.3. Assessing Nutritional Adequacy and Determining Cost Per Product

Using the nutrient content of individual product flavours, that average content of energy as well as macronutrients and micronutrients was computed for each of the product types available from that brand (e.g., shakes, soups, bars) and then combining three products per day proportionally to how the products would be expected to be consumed. For example, for brands that include shakes, soups and bars, we used one of each, but for brands that only include shakes and soups, we used two shakes and one soup, as in our experience most clients do not have more than one soup per day. The specific method for how the average daily content was calculated for each brand can be found in Table A1 in the Appendix A. The same method of combining products to determine average daily nutritional content was also used to determine the average cost per day, which was divided by three to obtain the average cost per product.

The average daily micronutrient content of each brand was compared against the Nutrient Reference Values (RDIs or where applicable, the adequate intakes [AIs]) for adult men and women aged ≥ 19 years [52]. The RDI is described as ‘the average daily dietary intake level that is sufficient to meet the nutrient requirements of nearly all (97%–98%) healthy individuals in a particular life stage and gender group’ [53]. Adequate intake is an alternate Nutrient Reference Value used when the RDI cannot be determined due to limited or inconsistent data and is described as, ‘the average daily nutrient intake level based on observed or experimentally-determined approximations or estimates of nutrient intake by a group (or groups) of apparently healthy people that are assumed to be adequate’ [53]. As there are no differences, or only small differences, between the RDI or AI for 19–70 years; and women aged > 70 years.

Low energy vegetables are included as part of many but not all VLED programs and would thus adults aged 19–30, 31–50 and 51–70 years [53], we collapsed them into one age group of 19–70 years. Where differences in RDIs or AIs within these age categories do exist, no matter how small, these are noted in the footnotes of respective tables. Therefore, the average daily content of each brand was compared against the RDI or AI of 4 groups: men aged 19–70 years; men aged > 70 years; women aged contribute to the nutritional content of VLEDs. However, as the nutritional contribution will vary greatly depending on which types and amounts of vegetables are consumed, and given that the majority of the population does not consume the minimum quantities of vegetables as part of their usual diet [52], and that—in our clinical experience—not all people who are prescribed low energy vegetables as part of a VLED consume them, the nutritional contribution of the low energy vegetables would be highly variable and were thus not included in the present analysis. However, the importance of their inclusion in VLEDs in the context of the nutritional content of the VLED products is discussed in Section 3.2 (Average Daily Macronutrient Content) and Section 3.4. (Average Daily Micronutrient Content)*.*

### 2.4. Rationale for Tailoring VLEDs to Estimated Protein Requirements of Individual Clients

Our secondary aim was to demonstrate ways in which protein content of prescribed VLEDs can be optimised at minimum cost, in order to provide guidance to clinicians on how to use and tailor VLEDs effectively for individual clients with varying protein requirements. In Australia, for adults aged 19–70 years, the RDI is 0.84 g/kg for men and 0.75 g/kg for women [45]. For adults aged > 70 years, the RDI is 1.07 g/kg for men and 0.94 g/kg for women [45]. As protein is the only nutrient for which the RDI varies according to body weight [53], the protein content of a VLED is the primary factor for which a VLED should be tailored. The National Health and Medical Research Council (NHMRC) Clinical Practice Guidelines for the Management of Overweight and Obesity in Adults, Adolescents and Children in Australia recommends the use of VLEDs in adults with BMI > 30 kg/m^2^, or with BMI > 27 kg/m^2^ and obesity-related comorbidities [3]. Therefore the body weight, and thus protein requirements, of potential clients for which a VLED are indicated will vary considerably.

When calculating nutritional requirements for individuals with obesity, there are three ways to adjust for increasing body weight, two of which attempt to take into account the disproportionate increase in less metabolically active fat mass to fat free mass: by actual body weight, by ideal body weight (weight at a BMI of 25 kg/m^2^), or by an adjusted ideal body weight (weight at a BMI of 25 kg/m^2^ plus 0.25 kg for each kg above this weight) [54]. The recommendations for protein intake in Australia, as well is in the UK and the USA, are based on *actual* body weight and do not include any guidance on adjusting body weight for BMI status [53]. The literature contains scarce discussion or guidance on this subject. Research to date into the optimal protein level for preventing loss of fat free mass and the associated increase in appetite during weight loss in individuals with overweight and obesity have used *actual* body weight as a means of calculating protein intake [23,29,30,31,55,56]. However, the majority of research has generally only included individuals with average BMIs up to and including 35 kg/m^2^, with very little data on optimal protein levels in individuals with average BMIs over 35 kg/m^2^ [11,21,22,23,24,25,26,27,28,29,30,31,32,33,55]. Further, the majority of interventions where the protein content of a weight loss diet is studied have been in the context of diets involving moderate energy restriction (~30%–40% reduction from weight maintenance energy requirements) [11,21,22,23,24,25,26,27,28,29,30,31,32,33,55]. When an intervention involves severe energy restriction (~60%–70% or even greater reduction from weight maintenance energy requirements), as in VLEDs, consideration must also be given to the feasibility of consuming enough protein within a very low energy limit (see [57] for further information on the feasibility and design of dietary interventions that meet protein intake within the context of moderate and severe dietary energy restriction). On the other hand, if an ideal body weight is used to estimate protein requirements instead of actual body weight, this would mean that a person with a BMI of 27 kg/m^2^ and a person with a BMI of 50 kg/m^2^, for example, would be prescribed the same amount of protein, which may not matter for a person with a BMI of 27 kg/m^2^ but could fall short of requirements for a person with a BMI of 50 kg/m^2^. Therefore, in line with the current literature based on protein requirements using actual body weights, we have also used an *actual* body weight to calculate protein requirements for individuals with a BMI ≤ 35 kg/m^2^. For individuals with a BMI > 35 kg/m^2^, we have used an *adjusted* body weight, adding only 0.25 kg for each 1 kg increase above a BMI of 35 kg/m^2^. That is, adjusted weight = ((height^2^ × 35) + (0.25 × (current weight − (height^2^ × 35)). Using these criteria, we calculated the weight of 48 potential clients from our four comparison groups mentioned above using three heights (below average, average and above average) and four BMIs (27, 30, 40 and 50 kg/m^2^). The average heights for men and women were taken from the 2011–2012 National Health Survey [58]. The below average and above average heights were chosen such that approximately 70% of the population would fall within the range of heights between the below average and the above average heights.

Using the body weights (actual or adjusted depending on BMI status) of these 48 potential clients, we estimated protein requirements using the following equation: estimated protein requirements (g/day) = weight (kg) × RDI (g/kg/day). We then calculated the difference between the average daily protein content of each brand and the estimated protein requirements for each of the 48 potential clients, to demonstrate the difference between the recommended level of protein and the quantity of protein provided by each brand. This information can then be used to tailor VLED protocols, as discussed in Section 3.3 (Strategies for Optimising the Protein Content of VLEDs).

## 3. Results and Discussion

### 3.1. Overview of Products Available

We identified eight brands of VLED products available in Australia; KicStart™ VLCD (KicStart™, Prima Health Solutions, Sydney, Australia), Optislim^®^ VLCD (Optislim^®^, OptiPharm Pty Ltd, Clayton, Australia), Optifast^®^ VLCD (Optifast^®^, Nestlé Health Science, Rhodes, Australia), Proslim VLCD, (Proslim, OptiPharm Pty Ltd, Clayton, Australia), Tony Ferguson^®^ VLCD (Tony Ferguson^®^, Tony Ferguson Weight Management Pty Ltd, Adelaide, Australia), Dr. MacLeod’s^®^ VLED (Dr. MacLeod’s^®^, Orfam Pty Ltd, Buderim, Australia), Cambridge^®^ Weight Plan (Cambridge^®^, Cambridge Weight Plan, Tullamarine, Australia), and Medical Vita Diet (Vita Diet, Vita Tech Pty Ltd, Molendinar, Australia). As shown in Table 1, all eight brands included shakes in their product ranges, seven included soups, five included bars, two included desserts and one included porridges. The total number of products available from each brand, including all of the different flavours, ranged from three for Tony Ferguson^®^ to 30 for Cambridge^®^. The average cost per product varied from $2.33 for KicStart™ to $4.43 for Cambridge^®^, which would result in a weekly difference of $44.10 if three products are consumed per day. Of note, Cambridge^®^ products are only available for purchase in Australia through Cambridge consultants and the higher price may reflect inclusion of a consulting fee, albeit there was no information on consulting fees on the Cambridge^®^ Weight Plan website in Australia. Products in powdered form require mixing with water or skim milk prior to consumption. It is important to take note of the manufactures’ instructions, as some products need to be mixed with skim milk to reach the nutritional content specified on the label. Mixing products with skim milk is not as practical as mixing products with water, especially for clients consuming the products away from home. Five brands included at least some options that were gluten free (Kicstart™, Optifast^®^, Dr. MacLeod’s^®^, Cambridge^®^ and Vita Diet). All options from three brands were free from artificial sweeteners (Kicstart™, Dr. MacLeod’s^®^, and Vita Diet), and one brand (Cambridge^®^) included some lactose free options. Although not entirely lactose free, the Optifast^®^ range includes several products in which the lactose content is low, at <1 g per serve. These findings are summarised in Table 1.

### 3.2. Average Daily Macronutrient Content

In line with our hypothesis, manufactures’ instructions generally expressed a “one size fits all” approach, whereby regardless of the size of the person, they are recommended to consume three products per day. There were three brands that proposed regimens containing three or more products per day (Optifast^®^, Dr. McLeod’s^®^ and Cambridge^®^), however only one of these brands (Optifast^®^) provided guidance on when this would be appropriate, with this information being available in the treatment protocol for clinicians [59]. However, even if it is not stipulated in the manufacturers’ recommendations, a clinician could advise (or a person could choose) the use of more than three products per day of any of the brands. Therefore, for the sake of comparability between brands, we have restricted our comparisons to consuming three products per day for all brands. Tailoring the VLED with the use of more than three products per day or other additions is discussed below, in Section 3.3 (Strategies for Optimising the Protein Content of VLEDs).

There was considerable variability in the average daily energy and macronutrient content of the different brands (Table 2). The average daily energy content ranged from 1765 to 2633 kJ. There was also significant variability in the average daily protein and carbohydrate content, ranging from 37 to 61 g for protein and from 34 to 80 g for carbohydrate. Presenting the average of three products is valuable because it demonstrates that although there may appear to be little difference between brands on a per product comparison, over a day small differences between products can result in a substantial difference. We noticed that choosing a brand with higher protein content would help to meet protein targets at lower levels of carbohydrate and energy, as well as cost. Specifically, comparing KicStart™ (the brand with the highest protein content) with a brand that contains less protein, Cambridge^®^ in this example, KicStart™ contains 8 g more protein and costs $2.10 less per product on average. This means that in order to reach a comparable protein intake of three products from the KicStart™ range (61 g), five products from the Cambridge^©^ range would be needed, resulting in an additional 31 g of carbohydrate, 677 kJ, and $15.16 per day.

In addition to energy, protein and carbohydrate content, it is important to consider the fat and fibre content of the different brands of VLEDs in order to minimise potential complications such as gallstone formation and constipation, respectively. There was large variability among brands in average daily fat and fibre content, ranging from 3–16 g for fat and from 1.3–12.4 g for fibre (Table 2). Five of the eight VLED brands (KicStart™, Optislim^®^, Optifast^®^, Proslim, and Dr. MacLeod’s^®^) shown in Table 2 contained an adequate total fat content (≥12 g/day) to minimise the risk of gallstone formation [60]. Information on essential fatty acids (linoleic acid and alpha (α)-linolenic acid) and long chain omega (Ω)-3 fatty acids was limited. Only Cambridge^®^ reported a combined value of linoleic acid and α-linolenic acid, and Optifast^®^ was the only brand to report Ω-3 fatty acids eicosapentaenoic acid (EPA) and docosahexaenoic acid (DHA); this was only in 7 out of 11 products. Thus, the considered evaluation of the fatty acid content of all of the VLED brands in comparison to the AI was not possible (Table 2). If VLEDs contain insufficient total or essential fatty acids, this can easily be remedied by adding small amounts (1–2 teaspoons/day) of oil to the recommended low energy vegetables [3]. While the addition of oil to the diet is not essential for most VLED brands, it does allow a greater flexibility and variety of dishes that can be prepared using low energy vegetables. The inclusion of low energy vegetables also helps to boost fibre intake and thus helps to prevent constipation, which is a common side effect of VLEDs [61], as well as helping with the social aspects of eating [4]. Inclusion into the VLED of low energy vegetables (typically two cups) was recommended by all but one of the VLED brands (Cambridge^®^). Some clients may still require a fibre supplement in addition to vegetables to prevent or alleviate constipation.

### 3.3. Strategies for Optimising the Protein Content of VLEDs

Table 3 compares the average daily grams of protein provided by each brand of VLED with the estimated protein requirements (in g/day) calculated using the RDI (g/kg/day) and weight (or adjusted weight where BMI > 35 kg/m^2^) of the 48 potential men and women of varying BMIs and heights aged 19–70 or > 70 years. In this potential population, weights used for the purposes of calculating estimated protein requirements ranged from 65 kg for a woman with a BMI of 27 kg/m^2^ and height of 1.55 m, to 133 kg (adjusted from 171 kg) for a man with a BMI of 50 kg/m^2^ and a height of 1.85 m. The estimated protein requirements for these individuals thus range from 49 g/day to 142 g/day (almost a 3 fold difference). Even KicStart™ and Optislim^®^, which have the highest protein contents at 61 g and 59 g per day, respectively, only meet the estimated protein requirements for women with the lowest requirements (i.e., smaller and younger women). There were no products that met the estimated protein requirements for any of the potential men of any age group included in our analysis. Demonstrating how the VLED brands compare to the estimated protein requirements for a range of potential clients is not intended to cause alarm or deter clinicians for using VLEDs. Indeed, VLEDs have been used safely in clinical practice for decades without apparent deleterious effects. However, we believe that there is a case for optimising the protein content of VLEDs based on an emerging body of work, particularly for older adults, which could potentially lead to better outcomes in terms of fat free mass preservation as well as helping with appetite control to aid adherence.

There are a number of ways we believe that VLED protocols could be optimised for protein intake for individuals with different nutritional requirements without product reformulation, with each method having advantages and disadvantages (Table 4). These are: supplementing with a pure powdered protein; supplementing with lean protein-rich foods (e.g., meat, chicken, fish, eggs); mixing the products with skim milk; and increasing the number of products used. In order to minimise additional energy and carbohydrate intake—which would reduce weight loss and could increase appetite—and avoid the temptation of food, we believe that the most suitable and cost effective option is to add pure powdered protein to powdered products such as shakes during preparation. A product that we use in our clinic and a current clinical weight loss trial [62] is Beneprotein^®^ (Nestlé Healthcare Nutrition, Rhodes, NSW, Australia). This product contains 6 g of protein and 104 kJ per 7 g scoop, no carbohydrate, dissolves readily in water and is relatively tasteless if less than two scoops are added per powdered product. If this product is used, six scoops can be added to three liquid products to boost daily protein intake by 36 g without the need for additional products. For example, using this formula of two scoops per product, protein intakes of up to 97 g/day can be achieved using three products from the KicStart™ range and six scoops of Beneprotein^®^; up to 130 g/day using four products of KicStart™ with eight scoops of Beneprotein^®^; and up to 161 g/day using five products of KicStart™ and ten scoops of Beneprotein^®^. This is also more cost effective than consuming additional products. Beneprotein^®^ is only $0.67 per 6 g of protein in a 7 g scoop (based on $21.95 for a 227 g can), and even adding 3 scoops to a VLED ($2.01 for 18 g protein) is cheaper than adding another product to the VLED, which—depending on the brand—would cost between $2.33 and $4.43 for 12–20 g protein. At the time of writing, we became aware of a product that has not yet been released on the Australian market but which may provide an alternative option. BODIE’z Protein Water (BODIE’z, Sydney, Australia) is set to release a line of carbohydrate-free, clear whey protein powders that dissolve completely in water and are also tasteless (according to the manufacturer’s website). However, these clear powdered whey protein products will be relatively expensive, at $100 for a 630 g tub, or $0.95 for 6 g of protein, compared to $0.67 for 6 g of protein from Beneprotein^®^. Being clear and apparently tasteless means that these products would not need to be added to powdered VLED products themselves, as they could be added to other liquids such as plain water or tea. BODIE’z also currently sell in Australia bottles of water (500 mL) containing 20–30 g of whey protein isolate and <1 g of carbohydrate, but these products are also relatively expensive compared to Beneprotein^®^, but not compared to the addition of another VLED product, at $5.80 per bottle ($1.16–$1.74 per 6 g of protein, depending on which variety is selected).

The two powdered protein product examples given above are both rich in whey proteins rather than the other major type of proteins found in milk (casein proteins), and this is likely to be important for reducing appetite. Whey and casein proteins differ in their chemical properties, which affect the rate at which they are digested and absorbed. This has given rise to the concept of ‘fast’ and ‘slow’ proteins for whey and casein proteins, respectively [63]. Casein proteins coagulate in the stomach due to precipitation by gastric acid, whereas whey proteins do not, resulting in relatively delayed gastric emptying for casein proteins [63]. Consequently, the post-prandial rise in circulating concentrations of constituent amino acids from casein proteins is less pronounced than that induced by whey proteins [63]. On the basis of Melinkoffs aminostatic theory of appetite regulation [64], a larger post-prandial rise in plasma amino acids is thought to increase satiety. Indeed, ingested whey proteins were found to result in higher post-prandial increases in circulating amino acid concentrations and greater satiety than ingested casein proteins in lean adults [65]. The satiating effect of whey proteins was further supported by greater increases in circulating concentrations of cholecystokinin and glucagon-like peptide-1 following ingestion, compared with casein proteins [65]. Therefore, adding whey protein powders to VLEDs may have a greater beneficial effect on appetite than adding casein protein powders.

In summary, whether a VLED diet regimen is supplemented with additional protein (ideally from whey), and how that protein is added, will need to take into account a variety of factors including client protein requirements, preference and cost.

### 3.4. Average Daily Micronutrient Content

The average daily micronutrient content provided by each of the VLED brands is shown in Table 5. No brand met the RDI or AI for all micronutrients, and therefore no brand could strictly be considered ‘nutritionally complete’ for all age and sex groups. This is in keeping with a 2009 report [66], which showed that neither of the two VLED brands that were included in both the current study and that study (KicStart™ and Optifast^®^) were found to be nutritionally complete for men or women (aged 35 years in the 2009 study). In the present study we include a further six brands of VLEDs that were not included in the 2009 report, as they were not yet available for purchase or not available at the pharmacies surveyed for the 2009 report. Information was not available for 10 micronutrients in the Vita Diet range, so that a considered evaluation of the micronutrient content of this product was not possible. Thus, it should not be considered suitable as a VLED as it is claimed to be on some products listed on the brands’ website. Below we highlight some micronutrients that may be of particular importance in certain clinical populations.

Folate and iron are particularly important nutrients in women of childbearing age [53]. Adequate intake of folate has been shown to prevent neural tube defects, most notably spina bifida in newborn babies [53], while iron is essential for transporting oxygen to tissues throughout the body, among other functions [53]. Only two brands of VLED (Tony Ferguson^®^ and Dr. MacLeod’s^®^) met (to the nearest µg) the RDI for folate for people of any age. Inclusion into the VLED of the recommended two cups of low energy vegetables will likely boost folate intake sufficiently, particularly with the inclusion of leafy green vegetables high in folate (e.g., spinach, broccoli, asparagus). As for iron, there were three brands of VLEDs (KicStart™, Cambridge^®^ and Vita Diet) that did not meet the RDI for iron for women aged 19–50 years. Given the likelihood that iron deficiency may already be present in women of this age [67], combined with the fact that the non-haem iron in VLED products is of lower bioavailability than haem iron from meat or other sources [53], and that VLEDs have been shown to lower iron status [68], iron status may need to be monitored in pre-menopausal women aged 19–50 years during use of a VLED. Although VLEDs are contraindicated during pregnancy [3], one study has shown that weight reduction associated with their use prior to conception (with a six-week period of re-feeding) improves fertility and reduces gestational complications in women with obesity [69]. Therefore, appropriate contraception during the use of a VLED and for at least six weeks after the diet should also be discussed.

Many individuals with obesity may have hypertension and/or be taking anti-hypertensive medications. Sodium and potassium both play key roles in blood pressure regulation [71]. While all of the brands of VLEDs met the AI for sodium (and were all also below the NHMRC Suggested Dietary Target of 1600 mg/day for the prevention of chronic disease [53]), none of the brands met the AI for potassium. Inclusion of the recommended two cups of low energy vegetables will boost potassium intake, but only by approximately 600 mg/day, which would still result in a shortfall in potassium intake of up to 1000 mg/day while on a VLED from the majority of brands. However, given the relatively low levels of sodium in all brands of VLED, as well as the fact that weight loss will also reduce blood pressure [72], the clinical significance of the low potassium levels in VLED brands is unlikely to be noticeable.

Older individuals have increased calcium and vitamin D requirements compared to their younger counterparts [48]. Both of these micronutrients are essential for multiple bodily functions, notably the maintenance of bone homeostasis [48]. None of the eight brands of VLEDs met the RDI for calcium for all age and sex groups, however all but two brands (Optifast^®^ and Cambridge^®^) met the RDI for those aged < 70 years. Similarly, while all brands met the AI for vitamin D for those aged < 50 years, only two brands (Dr. MacLeod’s^®^ and Vita Diet) met the AI for those aged > 50 years. Since diet-induced weight loss has been associated with a decrease in bone mineral density [58], and since obesity is associated with lower serum levels of 25-hydroxyvitamin D [59], supplementation of VLEDs with calcium and/or vitamin D may need to be considered in older individuals during winter months, or in individuals with a pre-existing deficiency.

Interpretation of the micronutrient content of the VLED brands and the clinical significance of these findings needs to be considered in the context of a person’s usual diet and current nutritional status. Individuals may be malnourished if their dietary intake of nutrients (vitamins and minerals) is low, even if their dietary intake of energy (kilojoules) is high [73,74]. Thus, given that some individuals with obesity may be consuming a diet of low nutritional value prior to a VLED [73,75,76], the micronutrient content of a VLED may actually be an improvement of a person’s usual diet, despite not providing 100% of the RDI. Further, VLEDs are typically consumed for relatively short durations of 8–12 weeks, whereas nutritional deficiencies may take many months to develop.

It is not known if the bioavailability of micronutrients from VLEDs is comparable to the bioavailability of micronutrients from food-based diets. Food factors, such as the matrix [77] and the potential for interactions amongst micronutrients, both synergistic and antagonistic, and the interactions between different components found in VLEDs, are factors that could influence micronutrient bioavailability [78]. In addition, physiological factors such as the effects of restriction in energy intake, may result in altered gastrointestinal nutrient absorption during VLEDs. Bioavailability studies of commercial weight loss products would help to address these questions.

In summary, although no VLED brand can be considered ‘nutritionally complete’ for all age and gender groups, due consideration should be given to the relatively low risk of developing nutritional deficiency diseases compared to the significant benefits gained from the loss of weight with a VLED in individuals with obesity. Given the lower than desirable levels of folate and potassium across the VLED brands, as well as fibre as mentioned in Section 3.2 (Average Daily Macronutrient Content), the importance of consuming the recommended two cups of low energy vegetables as part of a VLED should be emphasised.

### 3.5. Limitations and Further Information

In this paper we have proposed that prescribing VLEDs with added protein may improve adherence through better appetite control, while also attenuating loss of fat free mass during weight reduction. However, adherence to VLEDs is already relatively high, as evidence by the substantial and rapid initial weight loss achieved on average [5], as well as the over 80% success rate in losing 12.5% or more of initial body weight [6], albeit these weight loss figures do not indicate the amount of fat free mass loss. As such, adding protein to a VLED may not improve adherence further. Moreover, recent evidence suggests that the effect of protein to induce satiety may be reduced in older individuals [79], notwithstanding adequate protein intake is arguably more important in this population for attenuating loss of fat free mass and thereby helping to prevent exacerbation of sarcopenic obesity, if present. Additionally, appetite control is not the only factor affecting adherence. For example, the simplicity of a VLED program and the rapid weight loss associated with VLEDs may also be important motivators for adherence. However, apart from the added cost and preparation required, adding protein to a VLED in the form of a pure powdered protein does not have any foreseeable disadvantages, and may have advantages including better appetite control and body composition outcomes. Therefore, we believe that there is a case for adding protein to current VLED prescriptions.

A limitation of this work is that we only contacted and visited pharmacies in Sydney, Australia. Therefore, there may be other VLED products available in Australia that were not included in this report. However, we searched nationally-available websites and search engines, as well as contacting clinicians via a national interest group. We did consider calling pharmacies from across Australia in the process of conducting the study. However, it was decided that since the calls made to pharmacies in Sydney did not reveal any further brands that had not already been identified on national pharmacy websites, or through emails to specialist obesity dietitians practicing across Australia, this step would not be necessary. Therefore, we are confident that the brands included in this report are representative of those currently available throughout Australia. Another limitation of this work is that our analysis of the VLED products included in this report may not be generalizable to other countries. Nonetheless, the information hereby provided about tailoring VLEDs to diverse population groups is applicable globally. Finally, this paper is not intended as a standalone resource on the use of VLEDs. For example, physical activity and behavioural support (particularly for weight maintenance after VLED), paramount to the efficacy of VLEDs, are not discussed. Readers are referred to clinical practice guidelines and other associated resources for more detailed information on the use, side effects and contraindications of VLEDs [3,4,60,70,80,81,82].

## 4. Conclusions

This study has demonstrated the wide variability in the nutrient content, nutritional adequacy and cost of VLED brands available in Australia. These factors need to be taken into consideration when prescribing a VLED to a diverse range of potentially eligible populations in which these products are indicated, because a universal approach to VLED prescription is not always ideal. We propose that based on a body of evidence on the benefits of adequate protein intake during weight loss, VLED clinical treatment protocols should, at a minimum, meet protein (and micronutrient) requirements at the lowest carbohydrate and energy level, in order to help preserve fat free mass and control appetite (to aid adherence), while still allowing for rapid weight loss. We have discussed a number of ways in which this can be achieved with the products that are currently available on the market, without the need for reformulation. In order to meet protein requirements on a VLED while minimising additional carbohydrate, energy and cost, the most suitable option is to add a pure powdered whey protein.

## Figures and Tables

**Table 1:** Overview of types of products available, cost, preparation and suitability for special dietary requirements of brands of very low energy diet (VLED) products available in Australia. RRP, recommended retail price.

VLED brand characteristics	KicStart™	Optislim^®^	Optifast^®^	Proslim	Tony Ferguson^®^	Dr. MacLeod’s^®^	Cambridge^®^	Vita Diet
Number and type of products available	Total: 10 6 shakes 4 soups	Total: 11 5 shakes 3 soups 3 bars	Total: 14 6 shakes 3 soups 3 bars 2 desserts	Total: 14 7 shakes 3 soups 4 bars	Total: 3 3 shakes	Total: 9 5 shakes 2 soups 2 bars	Total: 30 12 shakes 7 soups 7 bars 1 dessert 3 porridges	Total: 7 5 shakes 2 soups
Average cost per product (based on RRP for largest quantity)	$2.33	$2.78 ^1^	$2.91	$2.80 ^1^	$2.97	$3.55	$4.43 ^1^	$3.00
Prepared with ^2^	Water	Water/skim milk (“Platinum” shakes only)	Water	Water/skim milk (“Rapid” shakes only)	Water	Water	Water or skim milk	Water
Suitability for special dietary requirements or preferences GF/ LF/AS	GF: yes (shakes only) LF: no AS: no	GF: no LF: no AS: yes (sucralose)	GF: yes (shakes and desserts only) LF: no (but the bars and desserts contain <1 g per serve) AS: yes (aspartame in shakes, bars and desserts)	GF: no LF: no AS: yes (sucralose)	GF: no LF: no AS: Yes (sucralose)	GF: yes (shakes only) LF: no AS: no	GF: yes (dessert and bars) LF: yes (6 options) AS: yes (aspartame)	GF: yes LF: noAS: no

^1^ This cost does not include the cost of the skim milk that is required for the preparation of some of the products in that brand. ^2^ Not applicable to bar products; AS, artificial sweeteners; GF, gluten free; LF, lactose free.

**Table 2:** Average daily energy and macronutrient content of very low energy diet brands available in Australia ^1^.

Energy or Macronutrient	KicStart™	Optislim^®^	Opifast^®^	Proslim	Tony Ferguson^®^	Dr. MacLeod’s^®^	Cambridge^®^	Vita Diet
Energy (kJ)	2633	2447	2517	2378	1765	2479	1986	2079
Protein (g)	61	59	54	53	53	49	38	37
Carbohydrate, total (g)	57	59	59	59	34	63	53	80
Sugars (g)	40	34	39	40	33	39	23	54
Fat, total (g)	12	15	14	13	5	16	10	2
Fat, saturated (g)	7.1	5.4	4.3	7.7	4	4.2	3.0	1.2
Essential fatty acids (linoleic acid & α-linolenic acid, mg)	N/A	N/A	N/A	N/A	N/A	N/A ^2^	3447	N/A
Ω-3 fatty acids (EPA & DHA, mg)	N/A	N/A	0–180 ^3^	N/A	N/A	N/A ^2^	N/A	N/A
Fibre (g)	12.0	1.4 ^4^	10.3	1.4	12.4	1.3	11.0	8.4

^1^ Average daily content was based on consuming three products per day. Detailed information about how this was calculated for each brand can be found in Table A1 in the Appendix A; ^2^ Quantitative information was not available, however website says it is a “good source of Omega 3 and Omega 6”; ^3^ Only in 6 out of 14 products; ^4^ Information missing for 7 out of 11 products and assumed to be 0. DHA, docosahexaenoic acid. EPA, Eicosapentaenoic acid; N/A, not available.

**Table 3:** Estimated protein requirements for a range of potential clients of different sex, age and BMI, and the difference (in g protein/day) from the average daily protein provided by very low energy diet brands available in Australia.

BMI (kg/m^2^)	Height (m)	Weight or Adjusted Weight (kg) ^1^	Estimated Protein Requirements (g Protein/Day) ^2^	KicStart™	Optislim^®^	Optifast^®^	Proslim	Tony Ferguson^®^	Dr. MacLeod’s^®^	Cambridge^®^	Vita Diet
**Women aged 19–70 years (RDI = 0.75 g/kg)**											
27	1.55	65	49	13	10	6	5	4	0	−11	−12
	1.62	71	53	8	5	1	0	0	−4	−15	−16
	1.70	78	59	3	0	−4	−5	−6	−10	−20	−22
30	1.55	72	54	7	4	0	−1	−1	−5	−16	−17
	1.62	79	59	2	−1	−5	−6	−6	−10	−21	−22
	1.70	87	65	−4	−7	−11	−12	−12	−16	−27	−28
40	1.55	87	65	−4	−7	−11	−12	−12	−16	−27	−29
	1.62	95	71	−10	−13	−17	−18	−18	−22	−33	−35
	1.70	105	79	−17	−20	−24	−25	−26	−30	−40	−42
50	1.55	93	70	−9	−11	−16	−17	−17	−21	−32	−33
	1.62	102	76	−15	−18	−22	−23	−23	−27	−38	−40
	1.70	112	84	−23	−25	−30	−31	−31	−35	−46	−47
**Women aged > 70 years (RDI = 0.94 g/kg)**											
27	1.55	65	61	0	−2	−7	−8	−8	−12	−23	−24
	1.62	71	67	−5	−8	−12	−13	−14	−18	−29	−30
	1.70	78	73	−12	−15	−19	−20	−20	−24	−35	−37
30	1.55	72	68	−6	−9	−13	−14	−15	−19	−30	−31
	1.62	79	74	−13	−16	−20	−21	−21	−25	−36	−37
	1.70	87	81	−20	−23	−27	−28	−28	−33	−43	−45
40	1.55	87	82	−21	−23	−28	−29	−29	−33	−44	−45
	1.62	95	89	−28	−31	−35	−36	−36	−41	−51	−53
	1.70	105	98	−37	−40	−44	−45	−45	−50	−60	−62
50	1.55	93	88	−26	−29	−33	−34	−35	−39	−49	−51
	1.62	102	96	−34	−37	−41	−42	−43	−47	−57	−59
	1.70	112	105	−44	−47	−51	−52	−52	−56	−67	−69
**Men aged 19–70 years (RDI = 0.84 g/kg)**											
27	1.70	78	66	−4	−7	−11	−12	−13	−17	−27	−29
	1.78	86	72	−11	−13	−18	−19	−19	−23	−34	−35
	1.85	92	78	−16	−19	−23	−24	−25	−29	−40	−41
30	1.70	87	73	−12	−14	−19	−20	−20	−24	−35	−36
	1.78	95	80	−19	−21	−26	−27	−27	−31	−42	−43
	1.85	103	86	−25	−28	−32	−33	−33	−37	−48	−50
40	1.70	105	88	−27	−30	−34	−35	−35	−39	−50	−51
	1.78	115	96	−35	−38	−42	−43	−43	−48	−58	−60
	1.85	124	104	−43	−46	−50	−51	−51	−55	−66	−68
50	1.70	112	94	−33	−36	−40	−41	−41	−45	−56	−57
	1.78	123	103	−42	−45	−49	−50	−50	−54	−65	−66
	1.85	133	111	−50	−53	−57	−58	−58	−63	−73	−75
**Men aged > 70 years (RDI = 1.07 g/kg)**											
27	1.70	78	83	−22	−25	−29	−30	−30	−35	−45	−47
	1.78	86	92	−30	−33	−37	−38	−39	−43	−53	−55
	1.85	92	99	−38	−40	−45	−46	−46	−50	−61	−62
30	1.70	87	93	−31	−34	−38	−39	−40	−44	−55	−56
	1.78	95	102	−40	−43	−47	−48	−49	−53	−64	−65
	1.85	103	110	−49	−51	−56	−57	−57	−61	−72	−73
40	1.70	105	112	−51	−54	−58	−59	−59	−63	−74	−75
	1.78	115	123	−62	−64	−69	−70	−70	−74	−85	−86
	1.85	124	133	−71	−74	−78	−79	−80	−84	−95	−96
50	1.70	112	120	−59	−61	−66	−67	−67	−71	−82	−83
	1.78	123	131	−70	−73	−77	−78	−78	−82	−93	−95
	1.85	133	142	−81	−83	−88	−89	−89	−93	−104	−105

Shaded areas indicate that the product meets the estimated protein requirements to the nearest 1 g; ^1^ Weights for potential clients with a BMI of 40 and 50 kg/m^2^ are the adjusted body weight; ^2^ Estimated protein requirements (g/day) were calculated using the RDI (g/kg/day) and weight (kg) (or adjusted weight were BMI > 35 kg/m^2^).

**Table 4:** Advantages and disadvantages of proposed strategies for increasing the protein content of very low energy diets.

Strategy	Advantages	Disadvantages
Supplementing with a pure powdered protein	Easy to administer (can be added directly to powdered products prior to preparation) Does not change taste considerably Relatively inexpensive Does not introduce extra food or meals No extra thinking around what to cook Does not change intake of other macronutrients or energy (can vary depending on type or brand of powdered protein used)	Adds extra cost (compared with not adding anything) Can make product thicker if additional liquid not also added Does not increase eating frequency Cannot be added to bars
Supplementing with lean protein-rich foods (e.g., meat, chicken, fish, eggs)	Clients feel like they are ‘eating’ something (chewing sensation) Can join in family meals Feels like a more ‘normal’ meal if combined with low energy vegetables Helps with social occasions Easy to buy and prepare Does not increase carbohydrate intake with low carbohydrate intake aiding in appetite control	Will increase energy and fat content Can be easy to overestimate quantity Can be expensive Have to think more about meals and what to cook Limited choice for vegetarians May reduce adherence [70]
Mixing the products with skim milk	Can improve taste (creamier mouth feel, sweeter) Easy to buy	Increases carbohydrate and energy content (which might affect appetite by reducing circulating ketone concentrations [46]): 200 mL of skim milk contains approximately 8 g of protein, 10 g of carbohydrate and has an energy content of 305 kJ Inconvenient when consuming products away from home (e.g., need to keep milk cold)
Increasing the number of products used	Easy to do Comes already mixed Only need to carry the product and not additional agents Little thinking involved Can provide a greater spread of intake over the day (i.e., more eating episodes)	More expensive (compared with other options) Also increases carbohydrate and energy content (which might affect appetite through reducing circulating ketone concentrations)

**Table 5:** Recommended dietary intake or adequate intake for micronutrients by age and sex, and comparison with the average daily micronutrient content of very low energy diet brands available in Australia.

Micronutrient	Sex and Age Group (Years)				KicStart™	Optislim^®^	Optifast^®^	Proslim	Tony Ferguson^®^	Dr. MacLeod’s^®^	Cambridge^®^	Vita Diet
	Men 19–70	Men >70	Women 19–70	Women >70								
**Recommended dietary intake (RDI)**												
Vitamin A (µg)	900	900	700	700	900	**792** ^1^	1214	**798** ^1^	**749** ^1^	1200	967	**795** ^1^
Vitamin C (mg)	45	45	45	45	60	56	113	53	75	84	117	60
Thiamine (mg)	1.2	1.2	1.1	1.1	1.7	1.4	2	1.3	2	2.4	1.5	1.7
Riboflavin (mg)	1.3	1.6	1.1	1.3	2.6	1.9	3	2.1	2	2.9	1.6	2.55
Niacin (mg)	16	16	14	14	**15** ^1^	21	23	21	19	21	18	**15** ^1^
Vitamin B6 (mg)	1.7	1.7	1.5	1.5	2.4	2.4	3	2.3	2	3.4	**1.6** ^1^	2.4
Biotin (µg)	30	30	25	25	**15**	**20**	101	**21**	201	185	57	N/A
Folate (µg)	400	400	400	400	**300**	**235**	**380**	**201**	399	460	**237**	**300**
Vitamin B12 (µg)	2.4	2.4	2.4	2.4	3	**2**	3	**1.7**	3	4	2.7	3
Calcium (mg)	1000	1300	1000	1300	**1200** ^3^	**1011** ^3^	**950**	**1170** ^3^	**1024** ^3^	**1200** ^3^	**854**	**1181** ^3^
Iodine (µg)	150	150	150	150	225	162	204	162	150	255	152	225
Iron (mg)	8	8	8–18 *	8	**14** ^4^	20	21	21	19	24	**15** ^4^	**17** ^4^
Magnesium (mg)	400–420 ^$^	420	310–320 ^¥^	320	480	**406** ^5^	**413** ^5^	438	361 ^1^	546	**375** ^5^	**390** ^5^
Phosphorus (mg)	1000	1000	1000	1000	1066	1001	1140	1183	**844**	1250	**882**	N/A
Selenium (µg)	70	70	60	60	**53**	**46**	82	**47**	60	79	**55**	N/A
Zinc (mg)	14	14	8	8	14	**12** ^1^	16	**12** ^1^	15	19	**10** ^a^	**9** ^1^
**Adequate intake (AI)**												
Pantothenic acid (mg)	6	6	4	4	**2.4**	**4.4** ^1^	9	**3.4**	8	**0.9**	6.8	N/A
Chromium (µg)	35	35	25	25	102	60	98	55	99	134	57	N/A
Copper (mg)	1.7	1.7	1.2	1.2	**1.5** ^1^	1.7	2	1.9	3	3	**1**	N/A
Manganese (mg)	5.5	5.5	5	5	**2.6**	**1.3**	**2**	**1.1**	**3**	**3.9**	**2.3**	N/A
Molybdenum (µg)	45	45	45	45	128	75	80	68	201	223	59	N/A
Potassium (mg)	3800	3800	2800	2800	**1547**	**1733**	**2267**	**1787**	**2088**	**1798**	**2116**	N/A
Sodium (mg)	460	460	460	460	929	1287	1177	1490	967	1384	1040	1141
Vitamin D (µg)	5–10 ^#^	15	5–10 ^#^	15	15	**8** ^2^	**7** ^2^	**9** ^2^	**9** ^2^	15	**6** ^2^	15
Vitamin E (mg)	10	10	7	7	15	13	19	13	12	17	13	15
Vitamin K (µg)	70	70	60	60	120	N/A	88	N/A	99	130	85	N/A

Bolded font indicates inadequacy for one or more sex and age groups.* 19–50 years 18 mg, 50–70 years 8 mg, ^#^ 19–50 years 5 µg, 50–70 years 10 µg, ^$^ 19–30 years 400 mg, 30–70 years 420 mg, ^¥^ 19–30 years 310 mg, 30–70 years 320 mg; ^1^ Does not meet the RDI for men only; ^2^ Does not meet the RDI for men and women aged >50 years; ^3^ Does not meet the RDI for men or women aged > 70 years only; ^4^ Does not meet the RDI for women aged 19–70 years only; ^5^ Does not meet the RDI for men aged >30 years only. N/A, not available.

## Appendix A

**Table A1:** Calculation of average daily composition and cost for each of the very low energy diet brands.

Brand	How the Average Daily Composition Was Calculated
Kicstart™	Average of all shakes Average of all soups Total: 2 average shakes + 1 average soup
Optislim^®^	Average of all shakes Average of all soups Average of all bars Total: 1 average shake + 1 average soup + 1 average bar
Proslim	Average of all shakes Average of all soups Average of all bars Total: 1 average shake + 1 average soup + 1 average bar
Optifast^®^	Average all shakes Average all bars Average all desserts Average all soups Total: [(1 average shake + 1 average bar + 1 average dessert + 1 average soup)/4] × 3
Tony Ferguson^®^	Average all shakes Total: 1 average shake × 3
Dr. MacLeod’s^®^	Average of all shakes Average of all soups Average of all bars Total: 1 average shake + 1 average soup + 1 average bar
Cambridge^®^	Average all shakes Average all bars Average all desserts Average all soups Average all porridges Total: [(1 average shake + 1 average soup + 1 average bar + 1 average dessert + 1 average soup + 1 average porridge)/5] × 3
Vita diet	Average of all shakes Average of all soups Total: 2 average shake + 1 average soup
