# Erratum: Gibson, A.A.; et al. Comparison of Very Low Energy Diet Products Available in Australia and How to Tailor Them to Optimise Protein Content for Younger and Older Adult Men and Women *Healthcare* 2016, *4*, 71

**DOI:** 10.3390/healthcare4040076

**Published:** 2016-10-12

**Authors:** Health Editorial Office

**Affiliations:** MDPI AG, St. Alban-Anlage 66, 4052 Basel, Switzerland

Please note that in the published paper [1], on page 5, three sentences in the second paragraph are wrongly placed in the third paragraph. The correct version is as follows:

“The average daily micronutrient content of each brand was compared against the Nutrient Reference Values (RDIs or where applicable, the adequate intakes [AIs]) for adult men and women aged ≥ 19 years [52]. The RDI is described as ‘the average daily dietary intake level that is sufficient to meet the nutrient requirements of nearly all (97%–98%) healthy individuals in a particular life stage and gender group’ [53]. Adequate intake is an alternate Nutrient Reference Value used when the RDI cannot be determined due to limited or inconsistent data and is described as, ‘the average daily nutrient intake level based on observed or experimentally-determined approximations or estimates of nutrient intake by a group (or groups) of apparently healthy people that are assumed to be adequate’ [53]. As there are no differences, or only small differences, between the RDI or AI for adults aged 19–30, 31–50 and 51–70 years [53], we collapsed them into one age group of 19–70 years. Where differences in RDIs or AIs within these age categories do exist, no matter how small, these are noted in the footnotes of respective tables. Therefore, the average daily content of each brand was compared against the RDI or AI of 4 groups: men aged 19–70 years; men aged > 70 years; women aged 19–70 years and women aged > 70 years.

Low energy vegetables are included as part of many but not all VLED programs and would thus contribute to the nutritional content of VLEDs. However, as the nutritional contribution will vary greatly depending on which types and amounts of vegetables are consumed, and given that the majority of the population does not consume the minimum quantities of vegetables as part of their usual diet [52], and that—in our clinical experience—not all people who are prescribed low energy vegetables as part of a VLED consume them, the nutritional contribution of the low energy vegetables would be highly variable and were thus not included in the present analysis. However, the importance of their inclusion in VLEDs in the context of the nutritional content of the VLED products is discussed in Sections 3.2 (Average Daily Macronutrient Content) and 3.4. (Average Daily Micronutrient Content).”

We apologize to the readers of *Healthcare* for any inconvenience.
